# The role of blood cell morphology in understanding and diagnosing severe fever with thrombocytopenia syndrome (SFTS): Insights from a case report

**DOI:** 10.1097/MD.0000000000040502

**Published:** 2024-11-08

**Authors:** Kailong Gu, Jin Gao, Lingyan He, Zengyun Lu, Yan Zhang

**Affiliations:** a Department of Clinical Laboratory, Hangzhou Xixi Hospital, Hangzhou Sixth People’s Hospital, Hangzhou Xixi Hospital Affiliated to Zhejiang Chinese Medical University, Hangzhou, Zhejiang, China; b Department of Clinical Laboratory, the Affiliated Hospital of Hangzhou Normal University, Hangzhou, Zhejiang, China.

**Keywords:** hemophagocytic lymphohistiocytosis, severe fever with thrombocytopenia syndrome, SFTS virus

## Abstract

**Rationale:**

Severe fever with thrombocytopenia syndrome (SFTS) is an emerging tick-borne zoonotic disease characterized by a high fatality rate of 12% to 15%. Despite hematologic abnormalities being frequently reported in SFTS patients, detailed characterization of peripheral blood cells, especially in severe cases, is scarce. This case report aims to fill this gap by describing the unique morphological features of blood cells in a patient with a severe outcome.

**Patient concerns:**

A male patient presented with severe symptoms of SFTS, including high fever and thrombocytopenia, and was subsequently infected with a highly virulent strain of the SFTS virus.

**Diagnoses:**

The patient was diagnosed with virus-associated hemophagocytic syndrome related to SFTS based on clinical presentation and laboratory findings.

**Interventions:**

The interventions included comprehensive supportive care and treatment aimed at managing the severe symptoms and complications associated with SFTS.

**Outcomes:**

Despite the interventions, the patient experienced significant morphological changes in white blood cells and ultimately succumbed to the illness.

**Lessons:**

The detailed description of abnormal cell morphology in this case provides valuable insights into the pathophysiology of SFTS. Recognizing these morphological abnormalities can aid in early detection and may have implications for assessing disease severity and guiding treatment strategies.

## 1. Introduction

Ever since the initial case of severe fever with thrombocytopenia syndrome (SFTS) was documented in China in 2011, extensive research has been conducted on the quantitative hematologic abnormalities associated with the disease.^[1–3]^ However, scant attention has been given to the specific characteristics of peripheral blood cells.^[4,5]^ In this report, we aim to shed light on the distinct features of morphological anomalies observed in circulating blood cells of a patient who was infected with a highly virulent strain of the SFTS virus (SFTSV).

## 2. Case presentation

A 79-year-old male farmer suffered from nausea, vomiting, and diarrhea for 8 days. Despite receiving treatment with Cefminox for infection, his condition worsened. He developed a persistent high fever, accompanied by tremors in his limbs and lips, slurred speech, and diarrhea. The consent forms and the proposed studies were approved by Institutional Review Board of Hangzhou Xixi Hospital.

### 2.1. Laboratory findings

The patient was admitted to our hospital, and he continued to experience a high fever. We observed nuchal rigidity, myoclonus in the lips and limbs, and increased muscle tone in his right lower limb. Leukopenia and thrombocytopenia persisted, and further tests revealed hematuria, proteinuria, and abnormalities in blood coagulation. The patient displayed elevated levels of aspartate aminotransferase (AST), alanine aminotransferase (ALT), lactate dehydrogenase (LDH), creatine phosphokinase (CK), creatine phosphokinase-MB isozyme, and ferritin, along with abnormalities in blood coagulation. Despite the presence of inflammation markers such as interleukin-6, procalcitonin, and serum amyloid A, C-reactive protein levels remained normal (Table 1).

**Table 1:** Laboratory data on the admission of our hospital.

Complete blood count (reference range)		Chemistry (reference range)	
White blood cell (3.50–9.50)	2.48 × 10^9^/L	Aspartate aminotransferase (15–40)	673 U/L
Red blood cell (4.30–5.80)	3.59 × 10^12^/L	Alanine aminotransferase (9–50)	108 U/L
Hemoglobin (130–175)	118 g/L	Lactate dehydrogenase (120–250)	1892 U/L
Platelet (125–350)	20 × 10^9^/L	Creatine phosphokinase (24–194)	548 U/L
**Coagulation system (reference range**)	134	Creatine phosphokinase-MB isozyme (0–25)	26 U/L
Prothrombin time (9.7–13.5)	20.2 s	Creatinine (57–111)	65 µmol/L
Activated partial prothromboplastin time (23–32.6)	113.1s	Blood urea nitrogen (3.6–9.5)	5.5 mmol/L
Thrombin time (14–21.0)	65.3s	Sodium (137–147)	133 mmol/L
Fibrinogen (1.8–3.5)	0.65g/L	Total bilirubin (3.42–20.52)	5.95 µmol/L
D–D dimer (0–0.55)	8.44mg/L	Total protein (65–85)	74.2 g/L
**Urinalysis**		Albumin (40–55)	15.6 g/L
Protein	++	Triglyceride (0.52–1.70)	1.99 mmol/L
Occult blood	+++	C-reactive protein (0–10)	6 mg/L
Sugar	+	Serum amyloid A (0–10)	114 mg/L
Leukocyte	+	Procalcitonin (0–0.500)	0.537 ng/mL
		Interleukin-6 (0–7)	47.97 pg/mL
		Ferritin (23.9–336.2)	1500 ng/ml
		Brain natriuretic peptide (0–125)	1633 pg/mL
		Troponin T (0–0.014)	0.021 ng/mL

On the second day of admission, the patient experienced a rapid decline in mental status, characterized by lethargy and unconsciousness. Consequently, an urgent transfer to the Intensive Care Unit was initiated for immediate treatment. The patient presented with a body temperature of 38.7 °C and required sedation due to severe agitation. Notably, the patient’s leukopenia exhibited dynamic fluctuations, with a white blood cell count of 2.66 × 10^9^/L, while thrombocytopenia reached a critical level, with a platelet count of 15 × 10^9^/L. The laboratory analysis revealed heightened levels of lactate dehydrogenase (6834 U/L), ferritin exceeding 1500 ng/mL, triglycerides measuring 3.82 mmol/L, AST of 1283 U/L, ALT of 128 U/L, blood urea nitrogen of 23.3 mmol/L, and serum creatinine of 202 μmol/L. Additionally, C-reactive protein was measured at 11 mg/L, procalcitonin at 4.700 ng/mL, and interleukin-6 at 6384.30 pg/mL.

Due to the patient’s manifestation of central nervous system symptoms, a cerebrospinal fluid analysis was performed, revealing slightly elevated protein levels and erythrocytosis. However, the results did not support the presence of an intracranial bacterial infection. Tests for Epstein-Barr virus, human immunodeficiency virus, cytomegalovirus, rubella virus, and herpes simplex virus yielded negative outcomes. The Weil–Felix test and Widal reaction also produced negative results. In light of the potential diagnosis of SFTS, the patient’s peripheral blood was sent to the Hangzhou Center for Disease Control and Prevention, where SFTSV positivity was confirmed through polymerase chain reaction detection (Ct value of 19, indicating a positive result if Ct value ≤ 38). Remarkably, the patient demonstrated 4 clinical indicators among the 8 diagnostic criteria for hemophagocytic lymphohistiocytosis (HLH)-2004,^[6]^ including fever, cytopenias, hypertriglyceridemia, hypofibrinogenemia, and an elevated serum ferritin level. Although a bone marrow examination was not performed, hemophagocytosis was observed in the blood smear. Furthermore, the patient exhibited elevated levels of transaminases, creatine kinase, creatine kinase isoenzyme, blood urea nitrogen, and hyponatremia. These findings strongly support the diagnosis of virus-associated hemophagocytic syndrome associated with SFTS. Regrettably, the patient’s condition continued to deteriorate, eventually leading to multiple organ failure.

### 2.2. Cell morphological characteristics

**Figure 1.**
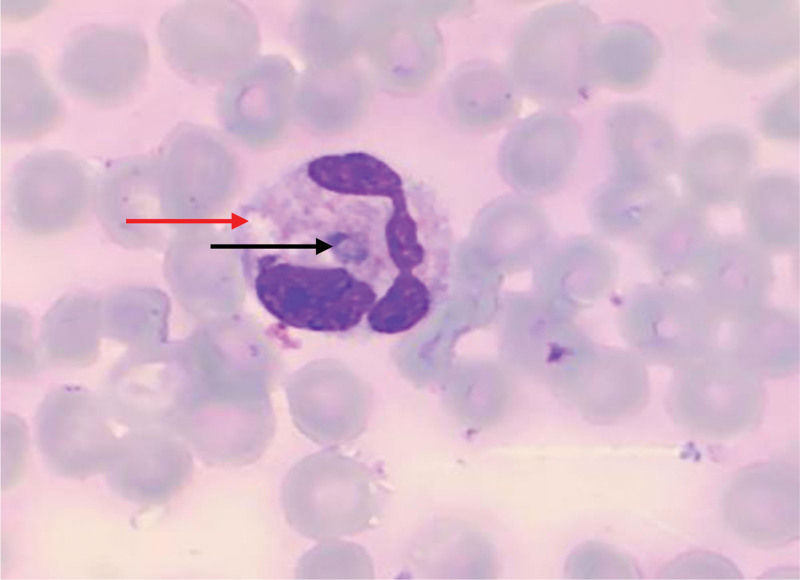
Neutrophil with vacuole (red arrow) and Döhle body (black arrow).

**Figure 2.**
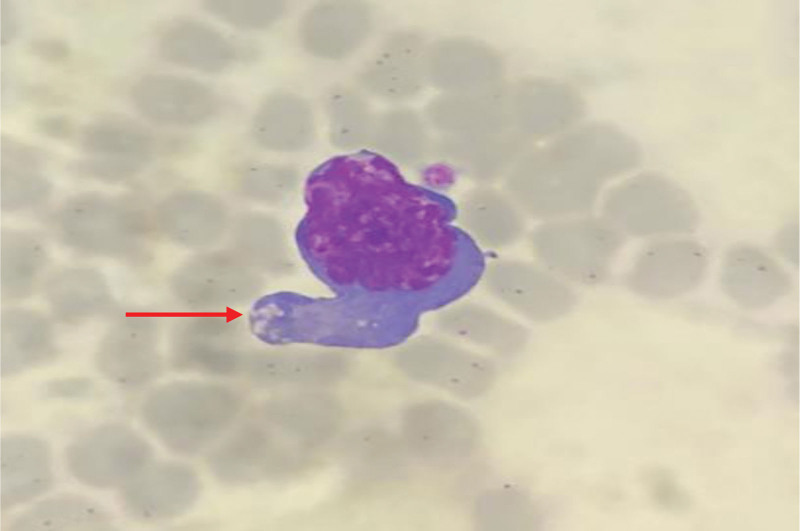
Atypical lymphocytes with hyperbasophilic and tail-like cytoplasm (red arrow).

**Figure 3.**
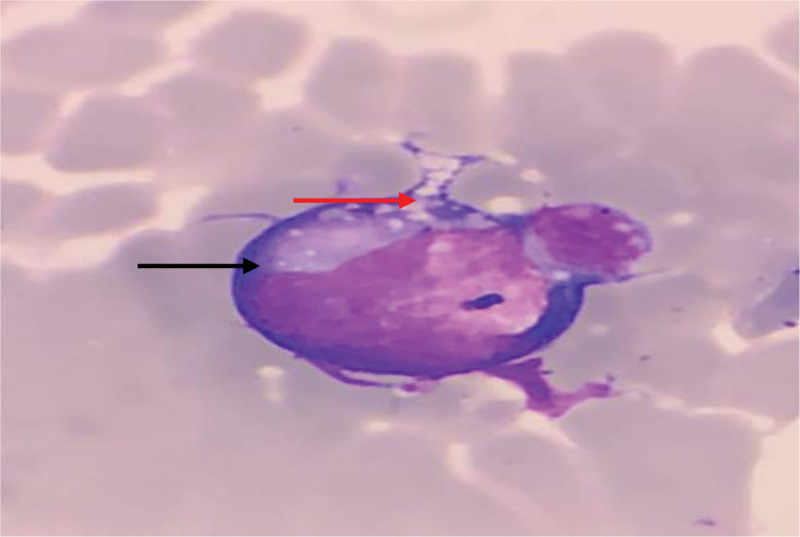
Atypical lymphocyte with vacuole (red arrow) and hyperbasophilic cytoplasm (black arrow).

**Figure 4.**
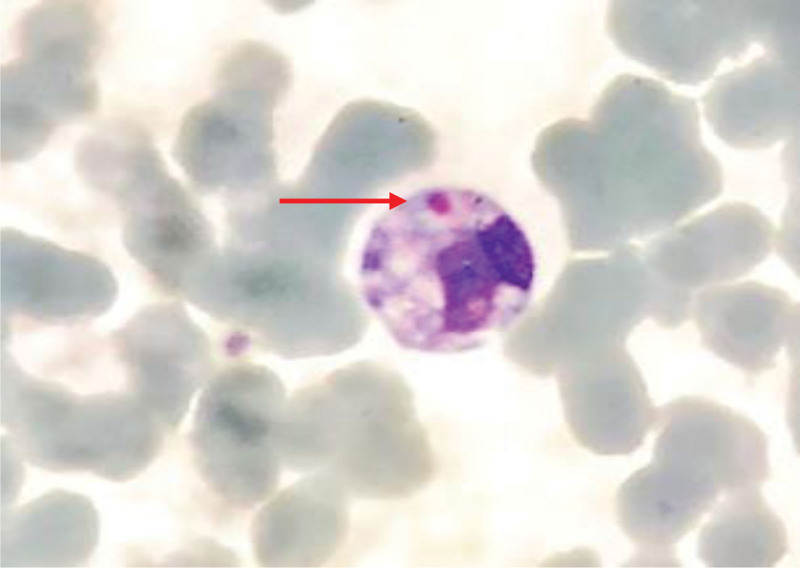
Activated monocytes phagocytosing a platelet (red arrow).

**Figure 5.**
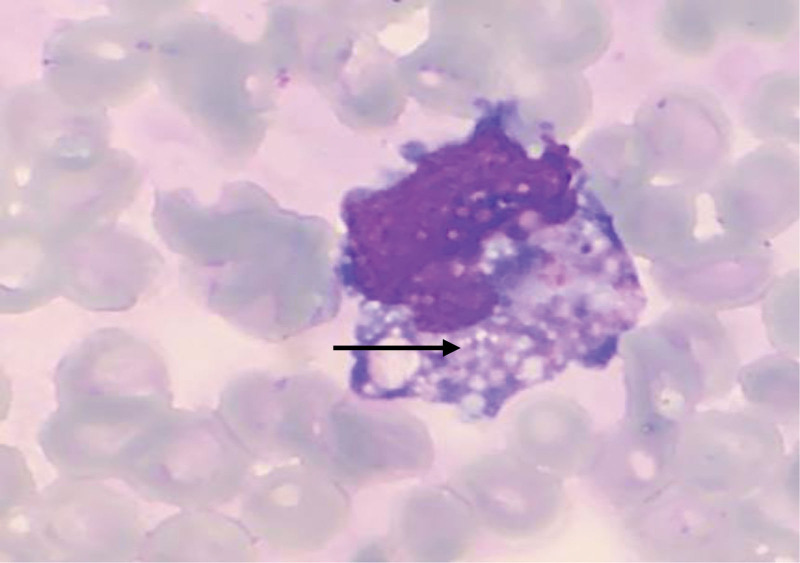
Activated monocyte with enlarged body and rich cytoplasm (black arrow).

**Figure 6.**
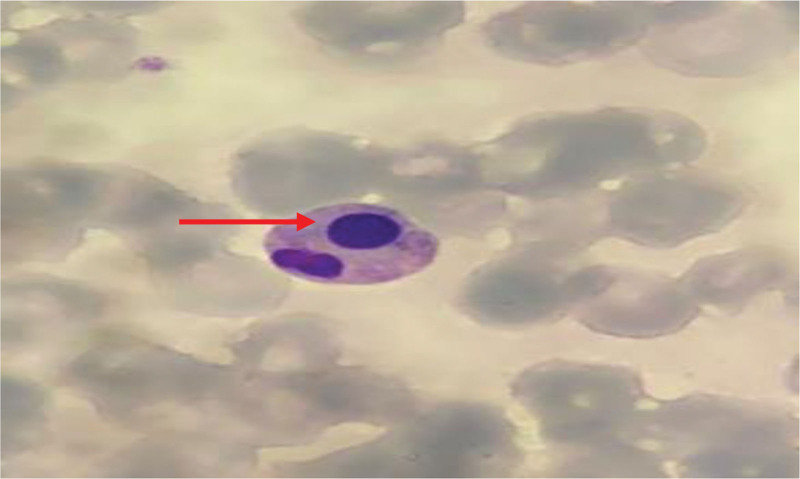
Apoptotic cell with fragmented nuclear chromatin (red arrow).

The peripheral blood smear revealed a condition characterized by an increased number of neutrophils (neutrophilic leukocytosis) with a neutrophil ratio of 82.5%. Morphological abnormalities were observed in both the nucleus and cytoplasm of these cells. Reactive changes, such as cytoplasmic vacuoles and Döhle bodies, were observed in certain neutrophils (Fig. 1). The lymphocyte population displayed significant morphological heterogeneity, including the presence of large atypical lymphocytes and lymphoplasmacytic cells. The proportion of atypical lymphocytes increased, ranging from 0.7% to 23.4%. These cells exhibited an increase in cytoplasm volume, with hyperbasophilic cytoplasm and some displaying tail-like extensions (Figs. 2 and 3). The monocytes were in an activated state, characterized by enlarged cell bodies with abundant cytoplasm. Phagocytic activity was also observed in some monocytes (Figs. 4 and 5). The presence of apoptotic cells was prominent in several peripheral blood films. These cells exhibited fragmented nuclear chromatin and granulated or deeply stained cytoplasm (Fig. 6). Additionally, the aberrant activation of macrophages further supports the involvement of the innate immune system in the pathogenesis of SFTS.

### 2.3. Treatment

The patient was given empiric anti-infective treatment with Shupu Deep 2.0 g Q8H in the early stage. However, the fever persisted and leukopenia and thrombocytopenia worsened. Considering the patient’s symptoms and signs, we upgraded the treatment to ceftriaxone 2.0 g Q12H, vancomycin 1.0 g Q12H, and acyclovir 0.4 g Q8H. Unfortunately, the patient’s condition progressed, he became unconscious, developed aspiration pneumonia, and suffered respiratory failure. He was transferred to the Intensive Care Unit and given endotracheal intubation and ventilator-assisted breathing, deep venous access, Shupu Deep 2.0 g Q8H anti-infection treatment, lansoprazole sodium acid suppression and stomach protection treatment. After treatment with hemostasis and furosemide injection to maintain diuresis, the patient’s condition did not improve. On day 6 of admission, the patient passed away.

## 3. Discussion

Patients diagnosed with severe fever with thrombocytopenia syndrome (SFTS) commonly present with various clinical manifestations, including high fever, gastrointestinal symptoms like nausea or diarrhea, bleeding, and severe central nervous system symptoms such as coma and convulsions.^[2,4]^ Laboratory tests typically reveal thrombocytopenia, leukopenia, and increased levels of certain enzymes in the blood.^[7]^ In this patient, levels of AST, ALT, CK, and LDH were significantly elevated, exceeding the normal range by approximately tenfold. Notably, significant elevations in AST, CK, and LDH serve as critical clinical indicators associated with a poor prognosis.^[8]^ Our study found that the patient exhibited these distinctive features, consistent with previous research.

Research on the cell morphology of SFTS patients is limited. Existing studies indicate that SFTS patients experience varying degrees of histiocytosis and hemophagocytosis, with some showing a significant increase in the proportion of mononuclear histiocytes.^[9]^Our findings align with these observations. Furthermore, we noted morphological changes in other peripheral blood cells of the patient. An increased proportion of neutrophils, along with the presence of cytoplasmic vacuoles and Döhle bodies, indicated systemic inflammation or a potential secondary infection. As the disease progresses, there is a tendency for a hematologic shift towards marked lymphocyte activation, often accompanied by an increase in their numbers and diverse morphological appearances. The SFTSV has the capacity to infect macrophages and B cell–lineage lymphocytes, leading to alterations in their morphology. Recent studies of fatal SFTS cases indicate that the virus primarily targets B cells differentiating into plasmablasts within tissues.^[10]^ Histopathological studies have revealed necrotizing lymphadenitis and significant hemophagocytosis as the pathological characteristics observed in fatal cases of SFTS.^[11]^ Apart from this, the virus can induce apoptosis directly in infected cells to facilitate its spread or inadvertently trigger cell sensors that initiate cell death. Morphological features of apoptotic cells include nuclear fragmentation, cell contraction, plasma membrane blistering, nuclear condensation and fragmentation, as well as the presence of apoptotic bodies.^[11]^ During microscopic examination of the patient’s peripheral blood cell morphology, we observed rounding, separation, and nuclear condensation in some cells (Fig. 6). In one particular cell, the nucleus was divided into 2 segments, and the cell’s atrophy was clearly evident from the increased nucleus-to-cytoplasm ratio. There is accumulating evidence suggesting that apoptosis induction plays a significant role in the pathogenesis of various viral diseases.^[12]^ The decrease in peripheral blood leukocytes and platelets in SFTS patients may be the result of the combined effects of SFTSV-induced bone marrow hematopoietic inhibition, cell maturation disorders, macrophage phagocytosis and apoptosis.

Overall, these morphological changes provide insights into the immune status of the deceased patients, highlighting the potential involvement of infectious or inflammatory processes that may have contributed to their mortality. These quantitative and qualitative abnormalities may be associated with the cytokine storm and hyperinflammation, possibly manifesting as secondary HLH, a condition that often leads to fatal multi-organ failure. As we known, secondary HLH is frequently associated with infections, autoimmune diseases, or malignant tumors.^[13]^ Most cases of secondary HLH are linked to viral infections, particularly in patients with Epstein-Barr virus infection, especially among Asians.^[14]^ In recent years, reports of SFTSV-related HLH have also increased.^[15,16]^The alterations in the patient’s cell morphology provided valuable insights. After ruling out a range of viral infections, including Epstein-Barr virus and cytomegalovirus, we conducted tests for the SFTSV, which ultimately returned positive. Consequently, we concluded that the patient was suffering from hemophagocytic syndrome induced by SFTSV. Hyperproduction of cytokines, including interferon γ, tumor necrosis factor α, and interleukin-6, by virus-infected T lymphocytes may significantly contribute to the pathogenesis of HLH. Infection-related hemophagocytic syndrome carries a high mortality rate, and the progression of SFTS is likely closely related to HLH secondary to the SFTSV.^[9,17]^

## 4. Conclusion

In summary, we presented a unique case of mortality due to SFTS and detailed the morphological characteristics of peripheral blood cells, including neutrophils, lymphocytes, monocytes, and apoptotic cells, alongside the associated secondary HLH, all of which were closely linked to the patient’s prognosis. Early recognition of these abnormalities, in conjunction with changes in clinical symptoms and hematological indicators, facilitates inferential and suggestive diagnoses, thereby promoting the timely detection of SFTS and offering valuable guidance for disease assessment and treatment strategies.

## Acknowledgments

We thank all study participants and staff of all participating sites.

## Author contributions

**Conceptualization:** Zengyun Lu, Yan Zhang.

**Data curation:** Jin Gao, Lingyan He, Zengyun Lu.

**Investigation:** Kailong Gu.

**Methodology:** Jin Gao.

**Project administration:** Kailong Gu.

**Writing—original draft:** Kailong Gu.

**Writing—review & editing:** Yan Zhang.
